# Extinction of the Influenza B Yamagata Line during the COVID Pandemic—Implications for Vaccine Composition

**DOI:** 10.3390/v14081745

**Published:** 2022-08-09

**Authors:** Zoltan Vajo, Peter Torzsa

**Affiliations:** Department of Family Practice, Semmelweis University Medical School, 1085 Budapest, Hungary

Vaccination remains the most effective way to mitigate the enormous burden of influenza on the health care system. Seasonal influenza vaccines routinely contain three or four virus strains, meaning trivalent or quadrivalent influenza vaccines, TIV or QIV, respectively. The components of the vaccines are based on the recommendations of the WHO and FDA’s Vaccines and Related Biological Products Advisory Committee, yearly or bi-annually. Recently, most human infections were caused by variants two influenza A strains—H1N1 and H3N2—as well as two influenza B strains—the Victoria and Yamagata lineages. However, the pandemic caused by the SARS-CoV-2 virus, and the imposed public health countermeasures, have resulted in some unexpected consequences. The Yamagata lineage of influenza B viruses has not been isolated since March of 2020, and is likely to be extinct by now [1]. In addition to the Yamagata lineage thought to be more vulnerable, and the domination of the Victoria lineage even prior to the COVID-19 pandemic; this is most likely resulting from the restrictions on travel and gatherings, as well as widespread mask use, since these limited the spreading of not only SARS-CoV-2, but also the various influenza strains.

The above will have obvious consequences on our vaccine strategies against influenza for the coming seasons. Since the Yamagata lineage of influenza B has not been detected for a prolonged period of time, vaccinating against it would make little or no sense. Consequently, one possible approach to influenza vaccine production would be to include only the three remaining strains currently causing human infections: Influenza A H1N1 and H3N2, as well as the Victoria lineage of influenza B viruses. This would increase the current production capability from approximately 500 million doses of QIV per year to 700 million doses of TIV per year instead.

All high-income, developed countries have wide recommendations for influenza vaccination, with the seasonal vaccine being recommended for most of the population; this is mandatory in some subgroups, such as health care workers in most cases [2]. These goals are hardly ever met, partly because of shortages occurring almost regularly [3]. In addition, developing countries suffer from distribution inequities as well as constant problems of vaccine shortage [4]. Hence, any means of increasing production capacity would be of enormous benefit, as influenza continues to cause millions of infections, hundreds of thousands of deaths, and billions of dollars lost due to health care costs and loss of working days. The final recommendations for TIV and QIV compositions and use, again, are made prior to each season by the WHO and FDA panels. 
